# New Features for Child Metrics: Further Growth References and Blood Pressure Calculations

**DOI:** 10.4274/jcrpe.galenos.2019.2019.0127

**Published:** 2020-06-03

**Authors:** Korcan Demir, Ergun Konakçı, Güven Özkaya, Belde Kasap Demir, Samim Özen, Murat Aydın, Feyza Darendeliler

**Affiliations:** 1 Dokuz Eylül University Faculty of Medicine, Department of Pediatric Endocrinology, İzmir, Turkey; 2 Ege University Faculty of Medicine, Department of Biostatistics and Medical Informatics, İzmir, Turkey; 3 Uludağ University Faculty of Medicine, Department of Biostatistics, Bursa, Turkey; 4 İzmir Katip Çelebi University Faculty of Medicine, Department of Pediatric Nephrology and Pediatric Rheumatology, İzmir, Turkey; 5 Ege University Faculty of Medicine, Department of Pediatric Endocrinology, İzmir, Turkey; 6 Ondokuz Mayıs University Faculty of Medicine, Department of Pediatric Endocrinology, Samsun, Turkey; 7 İstanbul University, İstanbul Faculty of Medicine, Department of Pediatric Endocrinology, İstanbul, Turkey

**Keywords:** Application, mobile, calculator, short stature, growth chart, hypertension, guideline, AAP

## Abstract

Many new features have recently been incorporated to ÇEDD Çözüm/Child Metrics, an online and freely accessible scientific toolset. Various auxological assessments can now be made with data of children with genetic diseases (Prader Willi syndrome, Noonan syndrome, Turner syndrome, Down syndrome, and Achondroplasia) and preterm and term newborns. More detailed reports for height, weight, and body mass index data of a given child are now available. Last but not least, office and 24-hour ambulatory blood pressure values can be analyzed according to normative data.

## Introduction

There exist various calculators for pediatricians generated by, but not limited to, the World Health Organization (WHO) (an offline tool for anthropometric calculations), UpToDate (online calculators for many specialties which requires a subscription), and individual developers including online and offline tools developed using Excel or Java Software) (1,2,3,4). In order to meet the specific needs of pediatric endocrinologists, we had launched an online and freely accessible scientific toolset containing a wide array of formulae under the official auspices of the Turkish Pediatric Endocrinology and Diabetes Society in 2017: ÇEDD Çözüm/Child Metrics (www.ceddcozum.com, www.childmetrics.org). In addition, the mobile application of Child Metrics can be downloaded from the App Store and Google Play. Currently, 550-600 daily users across various medical centers in Turkey work with the tool.

A description of the system was previously published in this journal (5). Briefly, standard deviation (SD) scores and percentile values can be calculated for weight, height, body mass index (BMI), and head circumference, using reference data from the Centers for Disease Control (CDC), Neyzi et al., and the WHO, as well as upper/lower segment ratio, waist circumference, sitting height/height ratio, IGF1 and IGFBP3 concentrations, growth velocity, bone mineral density, and thyroid and ovarian volumes. SD scores for a given measurement (x) are mainly calculated using LMS data with the following formulae: L≠0, SD score =[(x/M)**L–1]/LS or L=0, SD score=ln(x/M)/S (6). Interpolation by weighted mean is used to obtain L, M, and S values at finer intervals and that are not provided in the relevant references (7). When no LMS data are present for a variable, SD scores for a given measurement (x) are obtained by the following formula: SD score=(x–mean)/SD. Percentile values corresponding to calculated SD scores are obtained from a standard normal distribution table. In addition, various types of calculations for body surface area, target height, predicted adult height, growth hormone dose, tubular function tests, insulin resistance indexes, human chorionic gonadotropin test, and converting units of measurements are available (5).

With this review, we intended to present the newly added features and overview their scientific basis.

## Growth

### 1. Genetic Diseases/Syndromes

There are numerous benefits of specific growth charts for children with genetic diseases. Most importantly it is possible to assess the natural growth process for any genetic condition included. In addition, assessment and monitoring growth of affected children compared to peers with the same condition can be done. Inadequate growth according to syndrome specific curves would necessitate assessment for an associated comorbidity. On the other hand, some reference data might be biased, possibly due to relatively low numbers of cases included and variation in disease severity (8).

For Child Metrics, we selected the syndromes which are the most-relevant for pediatric endocrinologists. Key characteristics of the incorporated reference data are summarized in Table 1. Data are obtained from the published articles unless otherwise stated in the following sections. In the relevant section of Child Metrics, the measurements of the subjects are analyzed according to reference data of both healthy children and syndrome specific growth reference at the same time. When applicable, the results are plotted on specific electronic growth charts as well.

### *1.1. Prader Willi Syndrome*

In 2000, Hauffa et al (9) reported mixed cross-sectional and longitudinal data from German patients with genetically proven Prader Willi syndrome. Consequently, 123 data on height and 118 on weight and BMI were included in the analyses. They found no influence of genotypes or gender on SD scores of height, weight, and BMI.

Recently, Butler et al (10) published growth curves for both growth hormone-naive and -treated children with Prader Willi syndrome. In Child Metrics, we used the reference data belonging to white children who did not receive growth hormone. The majority of the measurements were obtained cross-sectionally. They noted that the height curves were found to be similar to previous German and USA graphs (9,10). The LMS data were obtained from Dr. Butler *via* personal communication.

### *1.2. Noonan Syndrome*

Ranke et al (11) published their mixed longitudinal and cross-sectional data in 1988 before the genetic diagnosis of Noonan syndrome was available. The data were collected retrospectively from the patient files of two medical centers with a long-standing interest in Noonan syndrome.

In 2012, Malaquias et al (12) reported reference data and growth curves for patients with pathogenic mutations in RAS/MAPK-related genes. The study included 137 patients (Noonan syndrome, n=119; Noonan syndrome with multiple lentigines, n=4, Noonan-like syndrome with loose anagen hair, n=4, and *CBL*-mutation associated syndrome n=10). Height and weight data were collected in a mixed longitudinal and cross-sectional method resulting in 536 observations. In each age group, approximately two-thirds of measurements were performed in children harboring *PTPN11* mutations. Among all genotypes, patients with *SHOC2* mutations were the shortest compared to subjects with other genotypes. The LMS data were obtained from Dr. Malaquias *via* personal communication.

### *1.3. Turner Syndrome*

The highest number of publications regarding condition-specific growth curves is in relation to Turner syndrome compared to other genetic diseases (13). We incorporated the widely accepted data of Ranke et al (14) published in 1983 and 1988. Among the included patients (n=150), 60% had 45,X karyotype. Reference data were generated in a mixed longitudinal and cross-sectional method.

### *1.4. Down Syndrome*

In their CDC-funded study published in Pediatrics in 2015, Zemel et al (15) reported growth charts for children with Down syndrome in the USA, mostly from the Philadelphia area. The majority of them were non-Hispanic white (73%). Researchers took a total of 1520 measurements from 637 individuals. Nearly two-thirds of subjects underwent measurement more than once and the average number of visits per subject was three (range, 1-9).

### *1.5. Achondroplasia*

Hoover-Fong et al (16) from the USA reported growth data at one-month intervals from 293 children with achondroplasia collected by a single observer between 1967-2004. Average numbers of height measurements per subject were 3.3 (range, 1-9) and 5.4 (1-22) among children below 3 years of age and between 2-16 years of age, respectively.

### 2. Detailed Reports

In addition to reporting centile and SD scores of height, weight, and BMI data, the following calculations are now made where available:

- Adult height in centimeter corresponding to current SD score of the given case.

- Height values in centimeters corresponding to -2, 0, and 2 SD scores and weight values in kilogram corresponding to the 3^rd^, 50^th^, 85^th^, and 95^th^ BMI centiles (equivalent to SD scores of -1.88, 0, 1.04, and 1.65, respectively) of given gender and age using the following formula: e^((ln((x)*L*S)+1)/L)+ln(M))^, where x is the desired SD score.

- BMI centile for height age of the given case, instead of calendar age (17).

- (If obese) Ratio of the BMI value of the given case to the 95^th^ centile of given gender and age (18).

## Newborns

We incorporated Turkish and USA reference data to assess length, weight, and head circumference of preterm and term newborns. Both data sets were based on the LMS method.

In 2012, Kurtoğlu et al (19) published their cross-sectional data collected retrospectively from the medical records of infants (n=4750, 52.5% male, 60.6% term) born at 28-42 weeks of gestational age during one year in 11 hospitals in Kayseri, a Central Anatolian city in Turkey. Infants whose mothers had chronic diseases, who were smokers or who had undergone multiple deliveries had been excluded, together with all infants who had fetal health problems, congenital malformations, and those with missing auxological data. Due to the low number of cases, some age groups were combined into the groupings 28-29, 30-31, 32-33 and 41-42 gestational weeks. The remaining data were given as per week of gestation. 

In 2013, Fenton and Kim (20) published their data, a combination of six large population-based surveys with different exclusion criteria. They were performed between 1991-2007 including 3,986,456 infants (34,639 births <30 weeks) from Germany, United States, Italy, Australia, Scotland, and Canada. The individual datasets were found to have good agreement with each other. The final LMS data were obtained from Dr. Fenton *via* personal communication. This dataset provides two different calculations according to gestational age input: (i) completed week (for weight: starting from 22 to 49 weeks, for length and head circumference 23-49 weeks), (ii) completed week + day (for weight: 22 weeks + 4 days to 50 weeks, for length and head circumference 23 weeks + 4 days to 50 weeks) (20).

### 3. Blood Pressure (BP)

BP values normally increase with age as the body grows; thus, comparing BP levels in mmHg among children are misleading. Instead, SD scores of office and ambulatory BP measurement (ABPM) values should be used.

### *3.1. Office Measurements*

**Figure 1:**
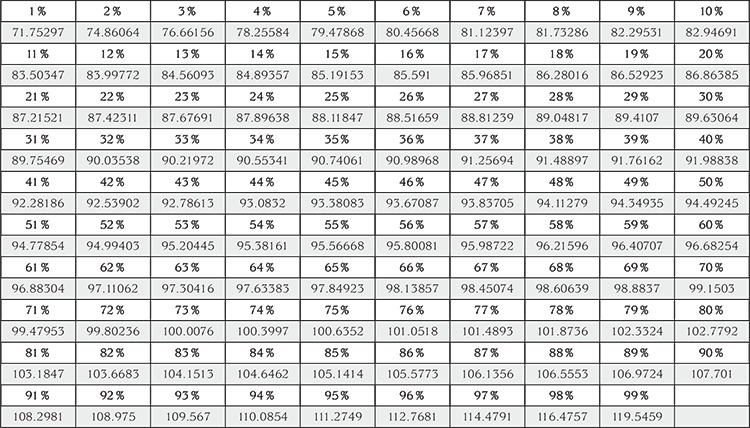
Reference centiles and corresponding systolic blood pressure values (mmHg) for an 8-year-old girl with a height of 123 cm generated by the system using relevant formulae and regression coefficients

In the 2017 Clinical Practice Guideline for Screening and Management of High Blood Pressure in Children and Adolescents, endorsed by American Academy of Pediatrics, detailed normative BP tables based on auscultatory measurements obtained from approximately 50,000 normal-weight children and adolescents (those with a BMI <85^th^ percentile between 1-17 years of age) are provided (21). Rosner et al (22) had published the methodology (quantile regression) used and a part of this normative data previously. For Child Metrics, equations and relevant regression coefficients were obtained from Dr. Rosner *via* personal communication. First, reference systolic and diastolic BP values corresponding to each of the 1^st^ through the 99^th^ centiles are generated for the given child using age, gender, and height/length data. Among these 99 reference values, the centile of BP that is closest to the child’s observed BP is reported. For example, a systolic BP of 95 mmHg corresponds to 52^nd^ centile for an 8-year-old girl with a height of 123 cm (Figure 1). The centile value is then converted to the corresponding SD score. The system also reports five BP values, decimals of which are omitted, corresponding to clinically relevant reference centiles, which indicate hypertension stages or target treatment thresholds: 50^th^, 75^th^, 90^th^, 95^th^ centile, and 95^th^ centile + 12 mmHg (21). The relevant BP values are 94 mmHg, 100 mmHg, 107 mmHg, 111 mmHg, and 123 mmHg, respectively, for the above-mentioned example.

### *3.2. Ambulatory Blood Pressure Measurements*

Before application of most ABPM devices, BP levels corresponding to 95th centile values according to gender and height should be entered. These data are most frequently obtained from the publications of Wühl et al (23) in 2002 and Flynn et al (24) in 2014. The data included in the latter article are reproduced based on the references reported by Wühl et al (23), which are generated with the LMS method. The articles provide sample reference BP data only for every 5 cm starting from 120 cm of height up to 175 cm for girls and 185 cm for boys; however, Child Metrics can provide relevant BP data for finer intervals based on the dataset presented by Wühl et al (23). As a result, individualized BP limits can be accurately established and while assessing the results of ABPM more proper BP loads may be calculated. In addition, SD scores of mean values of 24-hour, day, and night systolic, diastolic, and mean arterial pressure and centile values based on the same dataset can also be derived (23).

### 4. Future Agenda

We are working on solutions for analyzing multiple data (e.g. auxological data of 200 subjects) at once, creating growth curves including various data belonging to more than one visit, and increase the spectrum of IGF1 calculations by adding other types of kits available on the market.

## Figures and Tables

**Table 1:**
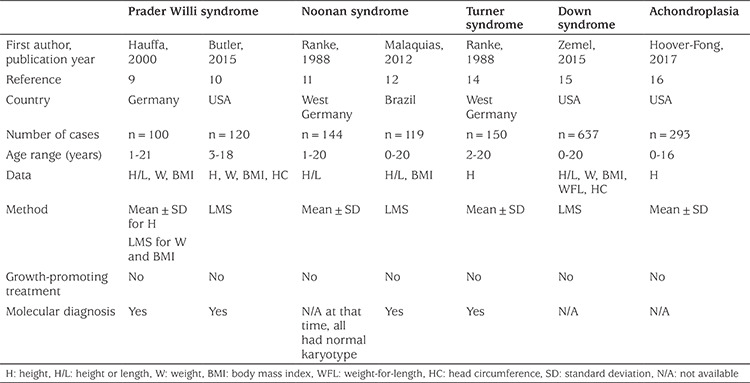
The characteristics of reference data for genetic diseases incorporated into Child Metrics
